# Bringing the Green Environment From Outdoors to Indoors Through Virtual Reality for Psychological Well-Being: Quasi-Experimental Study

**DOI:** 10.2196/93494

**Published:** 2026-09-16

**Authors:** Dorothy Bai, Jou-Ting Lin, Hao-Yun Huang, Hsin-Yen Yen

**Affiliations:** 1 School of Gerontology and Long-Term Care, College of Nursing, Taipei Medical University, Taipei, Taiwan, 886 2-2736-1661 ext 27749; 2 Department of Nursing, College of Medicine, National Cheng Kung University, Tainan, Taiwan; 3 Mater Hospital Brisbane, Queensland, Australia

**Keywords:** anxiety, depression, virtual reality, health promotion, urban health, environment exposure

## Abstract

**Background:**

Urban green spaces support psychological well-being, yet access is often limited for city residents. Virtual reality (VR) offers a promising indoor alternative, but evidence on sustained mental health effects of longer-term VR nature exposure remains limited.

**Objective:**

This exploratory pilot study aimed to explore the effectiveness of a 12-week VR green environment intervention in influencing self-reported psychological outcomes among urban residents.

**Methods:**

A nonrandomized control group pretest-posttest design was used with healthy urban adults aged 20 to 50 years. The experimental group received weekly 30-minute VR green environment sessions for 12 weeks, while the control group received no intervention. Self-reported psychological outcomes were assessed using standardized questionnaires and analyzed using generalized estimating equations.

**Results:**

Of the 60 participants recruited, 55 (91.6%) completed the study, with 30 (54.5%) in the experimental group and 25 (45.5%) in the control group. Significant group-by-time effects indicated that the experimental group showed reductions in distress (β=−5.17; *P*=.03), depression (β=−2.37; *P*=.003), anxiety (β=−3.20; *P*=.05), and somatization (β=−6.27; *P*=.004) scores, whereas the control group showed symptom deterioration. No serious adverse events were reported.

**Conclusions:**

This exploratory pilot investigation suggests that repeated VR green environment exposure has the potential to be a feasible health promotion tool for supporting psychological well-being among urban adults. These findings are preliminary and should not be interpreted as evidence of clinical therapeutic effectiveness.

## Introduction

Urban residents’ psychological well-being is a critical priority in modern public health [1]. The urban natural environment is a supportive health environment, especially for promoting psychological well-being [2,3]. Urban green environments provide health benefits for urban residents by mitigating air pollution, noise pollution, and heat, serving as potential pathways linking green spaces to health [4]. Urban residents may benefit psychologically when surrounded by a green environment, which has been associated with improved mood, reduced psychological distress, and better overall well-being [5,6]. However, there may be some obstacles and restrictions for urban residents to get close to a green environment every day, such as insufficient time, poor access to locations or transport connections, long distances, unfavorable weather or seasonal conditions, and institutional restrictions. It may be difficult for urban residents to find opportunities to visit green spaces for long periods [7].

Virtual reality (VR) provides an innovative approach to bring green environments from outdoors to indoors. VR can simulate visual and auditory stimulation of actual green environments. Users can feel that they are exposed to natural elements even in an indoor environment [8]. VR can mimic the natural environment by incorporating various natural elements that create a realistic and immersive nature experience. VR programs can provide access to nature by presenting users with virtual environments that simulate the sights and sounds of natural settings, such as forests or other landscapes [9,10].

VR, defined here as technology using head-mounted displays (HMDs) to provide immersive, 360° sensory experiences, brings nature indoors. Unlike desktop-based VR, HMD-based VR provides a higher level of presence by isolating the user from the physical room [9]. This includes using 360° video technology and high-quality visuals to recreate natural settings. To enhance the immersive natural experience, VR may integrate sensory information beyond visual and auditory inputs, including haptic, olfactory, or other tactile cues. These features have been proposed as ways to enrich the realism and multisensory quality of virtual nature [11]. Overall, VR mimics the natural environment by using advanced technology to create immersive and multisensory experiences that simulate natural settings, thereby providing individuals with access to nature [12].

The mental health benefits of VR nature involve multiple facets, with exposure to VR green environments mirroring the positive impacts of real-world nature exposure. It induces relaxation, reduces stress levels [11], and aids in managing anxiety and depression symptoms [13]. VR-based nature exposure enhances mood and positivity and captivates attention with resonating visual and auditory stimuli [12]. VR nature also holds promise in alleviating depressive symptoms associated with chronic ailments, fostering better psychological well-being by reducing rumination, and increasing mindfulness [11]. A VR natural experience can provide health effects similar to those of real natural experiences. Such experiences help users calm down, regain energy, reduce tension, improve fatigue, relieve stress, and enhance positive emotions [14]. VR provides sensory stimulation similar to that of actual natural environments, changes the connection between patients and nature, and creates a comfortable green space for interacting with nature for restorative, relaxing, and stress-reducing effects [15].

According to the existing literature on simulated green environments, exposure to virtual nature can alleviate symptoms of anxiety and depression and promote relaxation, ultimately improving psychological well-being [13]. Previous evidence-based interventions using simulated or restorative environments, including both standalone HMD-based VR and adjacent modalities (such as projection-based and laptop-based systems), suggested a substantial effect on improving multiple psychological outcomes, including anxiety [16,17], depression, stress [18,19], positive and negative affect [12], emotions [20,21], cognitive functions, and happiness [22].

However, there is still a research gap regarding VR green environments and psychological well-being. Previous interventional studies used VR green environments for rehabilitation training and for treating mental health diseases in health care settings, but they were seldom applied to healthy adult populations to assess long-term effects [23]. Most studies used only a single, short-term (5‐20 min) VR session to examine acute mental responses to VR green environments [19,24]. These changes may take time to manifest and solidify, necessitating prolonged intervention periods [9]. Additionally, the VR technology used in previous studies was also often outdated (ie, photos and 2D videos). Previous studies mainly investigated the effects of passive viewing or mouse-based navigation of VR nature scenes, while newer VR technologies allow for more immersive and interactive experiences [13].

The purpose of this exploratory pilot study was to explore potential effects of VR green environments on the psychological well-being of nonclinical urban populations. VR technology was used to bring green environments from outdoors to an indoor environment. The 12-week VR-based intervention via HMDs was designed with an experimental group (EG) and a control group (CG). The research questions were as follows:

- Did experiencing an intervention of a VR green environment have an effect on self-reported psychological indicators, including distress, anxiety, depression, and somatization, compared with those in the CG?
- Did experiencing an intervention of a VR green environment have an effect on participants’ positive and negative affect compared with that of participants in the CG?
- Did experiencing an intervention of a VR green environment have an effect on participants’ perceived stress compared with that of participants in the CG?

## Methods

### Study Design

This exploratory pilot study had a quasi-experimental design. A nonrandomized CG pretest-posttest design with one CG and a VR EG was conducted from September 2020 to June 2021. For clarity, this was a health promotion intervention study rather than a clinical trial, as the primary aim was to assess psychological well-being changes in a nonclinical cohort.

Participants were recruited through a website and by word of mouth among current participants. Students and residents near the university who were interested in VR could voluntarily join the study. Interested individuals could use a link or scan a QR code to access the registration form. After signing up, a research assistant would contact them to assess eligibility in the laboratory. Participants selected their respective groups based on personal preference and schedule availability. Data collection was conducted at 2 intervals: the pretest and posttest phases.

### Ethical Considerations

The study protocol was approved by the Taiwan Medical University Joint Institutional Review Board (N202002054). In accordance with ethical standards, written informed consent was obtained from all participants prior to their enrollment, following a comprehensive explanation of the experimental procedures. All participants joined the study voluntarily. To ensure data integrity and participant privacy, all collected information was anonymized and deidentified during the analysis phase.

### Participants

Participants were healthy adults with no disabilities who lived in an urban area and were interested in VR. “Healthy” was operationally defined as the absence of a self-reported history of a clinical mental health diagnosis or professional psychiatric treatment. No specific screening thresholds or clinical instruments were used for recruitment, as the study sought to examine variations within a nonclinical, general urban population. The inclusion criteria were participants aged 20 to 50 years who had not been hospitalized in the past year. Exclusion criteria included participants with any serious diseases, disabilities, or mental health problems that might influence the study process, those who had experienced severe VR sickness, and those who visited green spaces more than once a week. G*Power determined a minimum sample size of 34 for a repeated-measures, within-between interaction using the Four-Dimensional Symptom Questionnaire as the outcome, assuming a medium effect size (*f*=0.25), α=.05, and 0.80 power. A total of 60 participants were assessed for eligibility. All met the inclusion and exclusion criteria and consented to participate in the study. Participants were recruited until there were 30 in the CG and 30 in the experimental VR group. The study flow is illustrated in Multimedia Appendix 1.

### The Intervention

**Figure 1.**
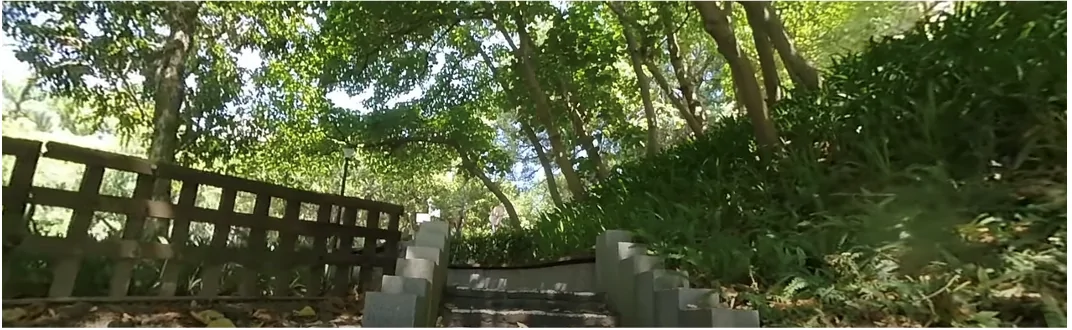
Example screenshot of virtual reality green spaces.

The intervention was designed to bring green environments from outdoors to indoors through VR. Videos of outdoor green environments were prerecorded in 360° format with a 360° camera (Insta360 EVO, Insta360) that was attached to a helmet to avoid anything blocking the front and back lenses. The locations of the outdoor environments in urban and suburban areas were chosen for their abundant green spaces, such as parks, hiking trails, mountains, and forests (Figure 1). All videos were recorded by walking or cycling along a route during the afternoon on a sunny day. A total of 12 series of monoscopic 360° videos featuring various green environments with natural elements were edited as interventional materials.

The EG received one 30-minute VR intervention session per week for 12 weeks. The study setting for an individual session was in a laboratory on the university campus without outside interference. No other people or extra noise or smells were allowed to disturb the session in the indoor environment. A research team member monitored all VR sessions. During each session, participants were asked to sit in a comfortable chair. Participants experienced the VR outdoor green environment by wearing an HMD for 30 minutes (Oculus Quest 2; Meta). The HMD was used to watch a 360° video and hear sounds using the YouTube (Google Inc) application. All participants watched a total of 12 different series of 360° videos in the same sequence during the VR sessions.

The intervention was designed as a passive viewing experience, meaning participants could not interact with virtual objects or move within the space using controllers. However, the 360° format provided 3 df, allowing users to explore the landscape through head movements. This approach simulated the restorative experience of “sitting and observing” nature, minimizing cognitive load and facilitating effortless attention. Participants were allowed to change their sitting posture or move their bodies slightly as long as they felt comfortable, but they were asked not to do any activities other than watching the VR video during a session. Participants were told to avoid insufficient sleep, excessive exercise, and consuming too much food or coffee before the session to avoid VR sickness.

Participants in the CG had no intervention during the 12 weeks. CG participants were instructed to maintain their daily routines and typical nature exposure during the 12-week study while refraining from using other VR technologies. To ensure the control condition represented a true baseline of urban living, participants had no contact with the research team between the pretest and posttest assessments.

### Outcome Measures

Self-reported psychological outcomes were collected using structured questionnaires at the pretest and posttest assessments. In addition, participants’ characteristics at baseline were collected, including age, sex, occupation (student and nonstudent), alcohol consumption (yes or no), smoking (yes or no), and chronic diseases (yes or no).

#### Psychological Well-Being

The 50-item Four-Dimensional Symptom Questionnaire with a 5-point scale was chosen for its ability to distinguish between normal distress and clinical symptoms in nonclinical populations. It provided scores for distress, depression, anxiety, and somatization, reflecting variations in psychological well-being. The questionnaire has demonstrated good content validity, criterion-related validity, and construct validity [25]. Cronbach α was 0.967 in this study.

#### Positive and Negative Affect

The Positive and Negative Affect Scale (PANAS) consisted of 50 items with a 5-point scale. The 2 subscales calculated the average scores for positive and negative affect. A higher score represented a higher perceived affective status. This scale was particularly sensitive to the mood-enhancing properties of nature exposure, which was hypothesized to increase positive affect and buffer against negative emotions by providing a restorative sensory VR experience. The PANAS has demonstrated good reliability and construct validity [26]. Cronbach α was 0.886 in this study.

#### Perceived Stress

Perceived stress was assessed using the Perceived Stress Scale (PSS), which consisted of 14 items with a 5-point scale. A higher score represented higher perceived stress in the past month. The PSS was included as VR nature exposure was theorized to facilitate stress recovery by reducing arousal through immersive stimuli. The PSS has demonstrated good reliability and criterion-related validity [27]. Cronbach α was 0.700 in this study.

### Statistical Analysis

Chi-square tests were performed to compare participants’ characteristics between the CG and EG, and Cramér *V* was calculated for the effect size. Independent 2-tailed *t* tests were performed to compare participants’ age and outcomes at baseline between the two groups, and Cohen *d* was calculated for effect sizes. The mean difference and 95% CI were calculated for differences between scores in the pretest and posttest in each group. Generalized estimating equations (GEEs) were used to analyze the group effect and group-by-time interactions on outcomes to answer the 3 research questions. The GEE analyses were adjusted for participants’ age and mental health scores in the pretest. SPSS (version 18.0; IBM Corp) was used for all statistical analyses.

## Results

Participants’ characteristics and outcomes at baseline are presented in Table 1. Sex, alcohol consumption, smoking status, and chronic diseases did not differ significantly between the EG and CG. However, participants’ age (*P*<.001) and occupation (*P*<.001) and several outcomes at baseline, including distress (*P*=.02), depression (*P*=.04), somatization (*P*=.008), and positive affect (*P*=.04), exhibited significant differences between the two groups. These results suggested that the two groups were heterogeneous. Therefore, participants’ age and scores at baseline were used for adjustment in the following GEEs. Ultimately, 30 participants in the VR group and 25 participants in the CG completed the intervention and the postintervention measurement.

**Table 1.** Participants’ characteristics and outcomes at baseline.

Variables	Experimental group (n=30)	Control group (n=30)	Chi-square (*df*)	*t* test (*df*)	*P* value	Cramér *V*	Cohen *d*
Sex, n (%)			2.78 (1)	—^a^	.10	0.219	—
Male	15 (50)	10 (33.3)					
Female	15 (50)	20 (66.7)					
Occupation, n (%)			27.15 (1)	—	<.001	0.673	—
Student	27 (90)	7 (23.3)					
Other	3 (10)	23 (76.7)					
Alcohol use, n (%)			0.01 (1)	—	.94	0.010	—
No	19 (63.3)	18 (60)					
Yes	11 (36.7)	12 (40)					
Smoking, n (%)			0.95 (1)	—	.33	0.128	—
No	29 (96.7)	30 (100)					
Yes	1 (3.3)	0 (0)					
Chronic disease, n (%)			1.96 (1)	—	.16	0.181	—
No	29 (96.7)	26 (86.7)					
Yes	1 (3.3)	4 (13.3)					
Age (y), mean (SD)	32.57 (8.72)	23.83 (2.23)	—	5.15 (58)	<.001	—	1.372
Distress, mean (SD)	26.79 (10.13)	21.67 (5.03)	—	2.41 (58)	.02	—	0.640
Depression, mean (SD)	8.50 (4.72)	6.50 (0.90)	—	2.21 (58)	.04	—	0.589
Anxiety, mean (SD)	16.43 (6.55)	14.27 (3.73)	—	1.53 (58)	.13	—	0.406
Somatization, mean (SD)	24.64 (7.47)	20.20 (3.85)	—	2.82 (58)	.008	—	0.747
Positive affect, mean (SD)	2.65 (0.75)	3.06 (0.72)	—	−2.09 (58)	.04	—	0.549
Negative affect, mean (SD)	1.99 (0.71)	2.04 (0.69)	—	−0.26 (58)	.80	—	0.067
Perceived stress, mean (SD)	24.83 (6.72)	24.73 (5.77)	—	0.06 (58)	.95	—	0.016

a Not applicable.

During each session, researchers systematically monitored participants for minor VR-related symptoms via verbal check-ins during and immediately following exposure. If participants experienced a headache, nausea, vomiting, or fatigue during the session, they could temporarily close their eyes and stop the video at any time. While a few participants reported mild, transient VR-related symptoms during the initial sessions, these symptoms resolved quickly without intervention. No participants requested to turn off the VR during any session. No serious adverse events were reported, and no participants discontinued the study due to discomfort. All 30 participants in the EG completed all 12 scheduled sessions (100% adherence rate).

GEE results indicated a group effect and group-by-time interaction on outcomes after adjusting for participants’ age and scores at baseline (Table 2). After the 12-week intervention, participants in the VR group exhibited decreased distress (β=−5.17; *P*=.03), depression (β=−2.37; *P*=.003), anxiety (β=−3.20; *P*=.05), and somatization (β=−6.27; *P*=.004), while participants in the CG exhibited increased distress, depression, anxiety, and somatization, with significant group-by-time interactions (*P*=.03, *P*=.003, *P*=.05, and *P*=.004, respectively). Participants in the VR group exhibited increased positive affect, while participants in the CG exhibited decreased positive affect, with a significant group effect (*P*<.001). All significant effects had a small effect size within each group (Cohen *d*≤0.03). Negative affect and perceived stress showed no significant effects.

**Table 2.** Results of the generalized estimating equation.

Outcomes	Experimental group			Control group			Group effect		Group-by-time interaction	
	MD^a^ (95% CI)		Cohen *d*^b^	MD (95% CI)		Cohen *d*	*B*	*P* value	β	*P* value
Distress	−0.17 (−1.86 to 1.53)		0.15	1.43 (−2.64 to 5.51)		0.04	0.05	.99	−5.17	.03
Depression	−0.30 (−0.90 to 0.30)		0.30	1.09 (−0.50 to 2.67)		0.19	0.37	.64	−2.37	.003
Anxiety	0.37 (−0.50 to 1.23)		0.15	1.04 (−1.93 to 4.01)		0.16	1.04	.51	−3.20	.05
Somatization	−0.77 (−2.58 to 1.05)		0.26	1.52 (−1.02 to 4.06)		0.16	1.82	.41	−6.27	.004
Positive affect	0.10 (−0.18 to 0.37)		0.03	−0.02 (−0.28 to 0.24)		0.13	0.91	<.001	−0.50	.10
Negative affect	0.09 (−0.14 to 0.33)		0.41	0.00 (−0.26 to 0.26)		0.15	0.27	.25	−0.22	.27
Perceived stress	0.33 (−1.79 to 2.46)		0.41	3.15 (0.02 to 6.29)		0.06	1.15	.47	−1.31	.40

a MD: mean difference.

b Cohen *d* for effect sizes of paired *t* tests within each group.

## Discussion

### Principal Findings

This exploratory pilot study aimed to bring outdoor green environments indoors via VR to support the psychological well-being of healthy urban residents. Our findings suggest that repeated exposure to these simulated environments was associated with favorable variations in self-reported psychological indicators compared with the CG. These interaction effects were partly driven by worsening CG scores, likely due to external urban stressors. Rather than clinical symptom alleviation, these changes reflect subtle variations in a nonclinical population. Due to the study’s pilot design and nonrandomized allocation, these findings provide preliminary evidence of feasibility and well-being promotion rather than definitive proof of clinical efficacy.

Participants who experienced the 12-week VR green environment intervention exhibited improvements in self-reported psychological indicators, including distress, depression, anxiety, and somatization. Similar results were found in previous interventional studies. A randomized controlled trial that conducted a single VR session for 20 minutes concluded that VR nature had an effect on improving anxiety among middle-aged and older adults [15]. Another study applied a 360° panorama to create a relaxing environment. College students who received a single VR session exhibited reduced anxiety scores [28]. Another previous crossover trial created a VR experience of the natural environment for 10 minutes a day for 10 days. Patients with psychiatric disorders exhibited significantly increased relaxation, calmness, and cheerfulness and decreased distress, anxiety, and nervousness after the VR sessions [29]. Another interventional study applied 5 natural scenes in an HMD set in a single VR session for at least 20 minutes. After the session, older adults felt more calm, more relaxed, more energetic, and happier and less sad, upset, worried, and anxious [30].

After the 12-week VR green experience, participants in the EG had increased positive affect. Similar results were found in previous studies. An interventional study applied a single 5-minute walking session in a forest. The forest scenario improved college students’ moods [31]. A single 15-minute VR forest video session was conducted in a long-term care facility, and residents with dementia exhibited improved emotions after the intervention [20]. Another study used a crossover experimental design with 30-minute HMD-based VR intervention sessions, twice a week for 12 weeks. Similar results were found in that VR natural videos had a positive effect on improving participants’ affect [14]. However, some studies revealed a nonsignificant effect of simulated natural experiences on affect and emotions in healthy adults and patients with heart failure [32]. One possible reason might be that those studies only conducted a single intervention with a lower immersive level than with HMDs (ie, television or photos) for an acute effect. The short-term VR session did not achieve a sufficient dose to gain mental health benefits.

However, participants in the VR EG showed no improvement in stress. Several studies with a single 6-minute VR session via an HMD found significant changes in healthy adult participants’ physiological stress indicators, such as salivary cortisol, galvanic skin response, or blood pressure [33] and after a 5-minute session among college students [19]. Another study conducted a single 40-minute VR session via television for college students [34]. Another study conducted a VR intervention for patients with cancer during chemotherapy [35]. However, stress is a complicated experience that might be influenced by changes in physical, emotional, psychological, and environmental strains and life events [36]. Even though a VR green environment can create a relaxing atmosphere and natural experiences for urban residents, the original stressors are difficult to remove through VR interventions.

There are several limitations to this exploratory pilot study. First, as recruitment focused on individuals interested in VR, the sample may be subject to novelty or expectation bias, as volunteers likely held favorable attitudes toward immersive technology. Second, while chosen for feasibility, this nonrandomized, participant-driven allocation led to baseline heterogeneity between the EG and CG. This introduces significant selection bias and limits the strength of causal inferences. The initial characteristics and outcomes at baseline of the two groups lacked comparability. Despite adjustments made through the GEE analysis to account for these variations, the effect of the VR green environment might have been biased. Third, we only recruited 60 participants for the study, and 5 participants in the CG did not return for the postintervention measurement. The small sample size might be a problem. Fourth, the resolution of the 360° video was not clear enough. During the editorial process, the research team tried to increase the resolution. However, it was difficult for the HMD to display a resolution similar to that of real green environments. The intervention used passive 360° videos rather than interactive environments. While reducing cognitive load, this lack of interactivity may limit the user’s sense of agency and active engagement, which are essential for high-immersion VR. Fifth, this study did not use a formal psychometric scale to quantify participants’ sense of presence or immersion. The lack of standardized immersion data limits the ability to correlate the level of presence with the magnitude of psychological improvement. Sixth, the CG had no intervention to control for time and attention during the weekly 30-minute sessions over the 12 weeks. The absence of an active control condition means that differences between groups may reflect novelty, expectation, or general study participation effects. Finally, this study only asked participants to maintain their regular lifestyles. Participants’ behaviors were not restricted during the study period, such as going to green spaces or engaging in more positive affect. Changes in outcomes in the EG were not guaranteed to have arisen solely from the VR intervention. This could introduce bias in interpreting the study results.

This study has practical implications. For health care practitioners, outdoor green environments are recommended to promote the psychological well-being of nonclinical urban residents. VR green environments are an innovative alternative for urban residents who do not have enough time to go outdoors, who have difficulties with access, who have a physical disability or limitations, and who live in a long-term care facility. Urban residents can experience an immersive green environment indoors through VR to gain mental health benefits similar to those gained outdoors.

This study also makes suggestions for future studies. An evidence-based study design with a randomized controlled trial could be conducted to examine the effect of VR natural environments. The sample size can be increased in diverse populations, such as populations with a sedentary lifestyle, older age, and physical limitations. The clarity of videos should be increased, and the weight of headsets should be reduced to avoid discomfort. The CG can receive an active control condition to ensure equal time and attention compared with the EG. For instance, participants can view identical 360° videos on computers or tablets to assess the level of immersion compared with VR. Finally, participants’ involvement in VR sessions can be monitored by eye trackers or facial recognition systems to ensure that they only focus on the 360° videos.

### Conclusions

VR technology is an efficient strategy to bring green environments from outdoors to indoors. VR can simulate urban green environments to create similar natural experiences with benefits for psychological well-being. This exploratory pilot study indicates that VR 360° nature exposure is a feasible health promotion tool that may influence psychological well-being in urban populations. Because of the heterogeneity between the EG and CG, it is advisable to interpret the study results cautiously in subsequent applications. Given the healthy status of the participants, these preliminary results should be interpreted as variations in psychological indicators within a nonclinical sample.

## Supplementary material

10.2196/93494 Multimedia Appendix 1 The study flow.

## Abbreviations

CG: control group
EG: experimental group
GEE: generalized estimating equation
HMD: head-mounted display
PANAS: Positive and Negative Affect Scale
PSS: Perceived Stress Scale
VR: virtual reality
