# Supplementary material for: Bringing the Green Environment From Outdoors to Indoors Through Virtual Reality for Psychological Well-Being: Quasi-Experimental Study
Source: J Med Internet Res. 2026 Sep 16;28:e93494. doi: 10.2196/93494 (PMC13581148; doi:10.2196/93494)

Figure S1 The study flow

CONSORT

TRANSPARENT REPORTING of TRIALS

CONSORT 2010 Flow Diagram

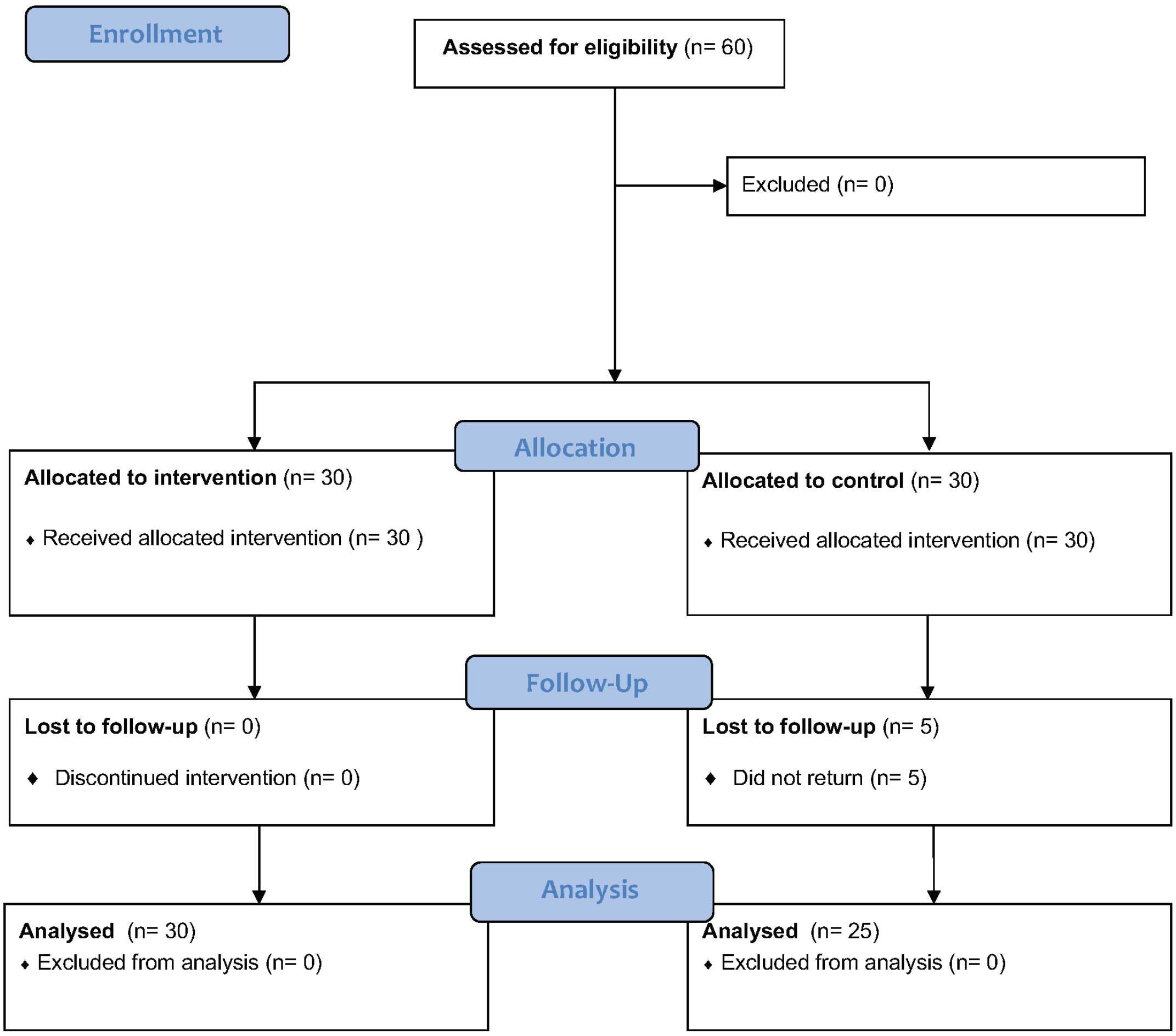

Supplement: Supplementary file 1 [file jmir-v28-e93494-s001.pdf]
